# Mutational analysis of highly conserved aspartate residues essential to the catalytic core of the *piggyBac* transposase

**DOI:** 10.1186/1471-2199-9-73

**Published:** 2008-08-11

**Authors:** James H Keith, Cheryl A Schaeper, Tresa S Fraser, Malcolm J Fraser

**Affiliations:** 1 University of Notre Dame, Notre Dame, Indiana, USA

## Abstract

**Background:**

The *piggyBac *mobile element is quickly gaining popularity as a tool for the transgenesis of many eukaryotic organisms. By studying the transposase which catalyzes the movement of *piggyBac*, we may be able to modify this vector system to make it a more effective transgenesis tool. In a previous publication, Sarkar A, Sim C, Hong YS, Hogan JR, Fraser MJ, Robertson HM, and Collins FH have proposed the presence of the widespread 'DDE/DDD' motif for *piggyBac *at amino acid positions D268, D346, and D447.

**Results:**

This study utilizes directed mutagenesis and plasmid-based mobility assays to assess the importance of these residues as the catalytic core of the *piggyBac *transposase. We have functionally analyzed individual point-mutations with respect to charge and physical size in all three proposed residues of the 'DDD' motif as well as another nearby, highly conserved aspartate at D450. All of our mutations had a significant effect on excision frequency in S2 cell cultures. We have also aligned the *piggyBac *transposase to other close family members, both functional and non-functional, in an attempt to identify the most highly conserved regions and position a number of interesting features.

**Conclusion:**

We found all the designated DDD aspartates reside in clusters of amino acids that conserved among *piggyBac *family transposase members. Our results indicate that all four aspartates are necessary, to one degree or another, for excision to occur in a cellular environment, but D450 seems to have a tolerance for a glutamate substitution. All mutants tested significantly decreased excision frequency in cell cultures when compared with the wild-type transposase.

## Background

With the successful application of the *Drosophila melanogaster P *element as a tool for the creation of transgenic fruit flies, researchers had high hopes for the stable introduction of transgenes into germlines [1]. Unfortunately, *P *element mobility was determined to be restricted only to *D*.*melanogaster *and very closely related species [2]. The ensuing search for novel mobile elements that are active within a wide range of species led to the classification and characterization of the *hAT*, *Tc1-mariner*, and *piggyBac *families of mobile elements [3]. The *piggyBac *transposon is a short repeat, class II mobile element originally isolated as the causative agent of the Few Polyhedra plaque morphology mutations of *Autographa california *nuclear polyhedrosis virus (AcNPV) [4]. It is the archetype of its own family and the only fully active transposable element in that family that is currently in use as a transgenic vector. The wild-type *piggyBac *element is 2.4 kb long and ends in a 5' CCC...GGG 3' configuration with asymmetric terminal and subterminal repeats. The sub-terminal repeats are 19 bp in length and lie 3 bp and 31 bp inside of the perfect inverted terminal repeats (ITRs) on the 5' and 3' end respectively [4]. The protein catalyzing the movement of *piggyBac *is encoded within a single open reading frame (ORF) of 1783 bp, with a length of 594 amino acids and a predicated mass of 68 kDa [4,5]. As a class II mobile element, *piggyBac *operates via a DNA intermediate in a cut-and-paste mechanism [6]. In this scenario, the transposable element, delineated by transposase-specific ITRs is excised by the transposase and reinserted into a new location. In the case of *piggyBac*, the bias for individual sites chosen during reinsertion does not follow any clearly defined rules. Although the only sequence requirement for a new insertion event is the presence of a TTAA sequence in the DNA, there is a clear preference for certain TTAA sites over others as shown by inter-plasmid transposition assays [7].

The *piggyBac *element has been used to transform the species *Mus musculus *[8], *Tribolium castaneum *[9], *Anopheles gambiae *[10], *Ceratitis capita *[11], *Drosophila melanogaster *[12], *Bactrocera dorsalis *[13], *Musca domestica *[14], *Lucilia cuprina *[15], *Bicyclus anynana *[16], *Aedes aegypti *[17,18], *Anopheles albimanus *[19], *Anopheles stephensi *[20], *Bombyx mori *[21], *Athalia rosae *[22], *Drosophila willistoni *[23], *Pectinophora gossypiella *[24], *Anastrepha suspensa *[25], *Aedes fluviatilis *[26], *Harmonia axyridis *[27], *Schistosoma mansoni *[28], and the causative agent of malaria, *Plasmodium falciparum *[29]. Plasmid-based mobility assays have also shown *piggyBac *to be active in human and other primate cells [8,30], in *Zea maize *cells [31], *Spodoptera frugiperda *cells [5], and in the embryos of *Aedes triseriatus *[32], *Heliothis virescens *[33], and *Danio rerio *[30], although none of these species have yet been transformed with *piggyBac*.

Recently, Mitra et al. [34] have defined *piggyBac *mediated transposition as involving several discrete steps. First, the transposase creates 3' nicks at each end of the terminal repeats just internal of the 'TTAA'. The free 3'-OH attacks the complimentary strand, four nucleotides downstream of the initial nick, just outside of the 'TTAA' repeat, forming a transient hairpin structure and releasing the transposon ends. The transposase quickly resolves these hairpin structures into 5' 'TTAA' overhangs on either side of the excision product. For reinsertion, the transposase joins the excised element to a new 'TTAA' target site in a staggered pattern, completing the transposition reaction. This mechanism accounts for both precise excision and target site duplication.

For all transposable elements, there are certain requirements for transposition, namely the recognition and binding of the terminal repeats, double stranded DNA breakage (DSB), and reinsertion of the element into a target, as well as possibly the repair of the excision site, which may or may not involve host factors [3]. DNA manipulating enzymes, such as integrases, recombinases, resolvases, endonucleases, and transposases must meet one or more of these requirements in order to successfully catalyze their respective reactions. With this commonality in function comes some similarity in mechanism and structure. One of the most widespread of these features is the 'DDE/DDD' motif first identified and designated in retroviral and retrotransposon integrases [35]. This motif has since been identified among many different families of DNA manipulating enzymes, from certain nucleases to the enzymes that carry out V(D)J recombination and even the human RNAi enzyme, Ago2 [36-47]. 'DDE/DDD' has been implicated as the active site for such enzymes by providing carboxyl groups used to recruit metal ions as essential cofactors. These residues are required to precisely position the metal ions, usually magnesium or manganese, used to recruit nucleophilic groups to the catalytic center of their respective enzymes.

Sarkar et al. published an extensive computational analysis of the *piggyBac *element and related sequences [48] that demonstrated the widespread occurrence of the *piggyBac *family of elements. While no actual experiments were performed by Sarkar et al., they proposed a 'DDD' motif for *piggyBac*, similar to a paper published in 1996 in which Bigot et al. speculated the existence of a 'DSE' motif in *hAT *elements based on sequence alignments. Sarkar et al. based their proposal on weak matches for two *piggyBac *related proteins to the bacterial *IS*4/5 transposase family DDE motif. This alignment suggested the first two of these aspartates are D268, and D346. While this similarity with the *IS*4/5 transposases did not yield a hit for the third member of the 'DDE' triad, Sarkar et al. speculated that the third member was an aspartate at D447.

In this report, we align very closely related members of the *piggyBac *family across kingdoms and identify several highly conserved regions. We propose that a direct relationship exists between the degree of conservation of a particular feature, and its necessity to catalyze transposition, as they are favored at the time of horizontal transmission. To this end, we only considered the proteins most closely related to *piggyBac *in our alignment. However, it should be made clear, when considering the results of our alignment, aside from *piggyBac *itself, only one other family member has been shown to be a functional element: Xtr-Uribo2_PCR_Iv1b (Genbank Accession: BAF82025) from the *Xenopus *family, which catalyzes movement in GP293 cells [49]. Therefore, we are careful to judge the relevance of any well conserved feature primarily upon its presence in either *piggyBac *or Uribo-2.

Finally, we mutate the three aspartates identified by Sarkar et al. as well as D450, another highly conserved residue close to the third proposed aspartate, which may also contribute to the catalytic center of the transposase. We test these site-directed mutant transposases for excision activity in two different donor plasmids and report our results.

## Results

### Sequence alignment

We performed a GenBank [50] search for proteins closest in similarity to the wild-type *Trichoplusia ni piggyBac *transposase sequence. We chose hits from a wide variety of organisms for a sequence alignment by ClustalW (Table 1). We then exported the sequence alignment file and entered it into the BoxShade server [51] to more easily visualize any conserved residues or groups of residues.

**Table 1:** Proteins used in the alignment

**Accession**	**Taxon**	**Protein Name**
J04364	Trichoplusia ni	piggyBac
AAM76341	Daphnia pulicara	Pokey 6.6 kb
AAM76342	*Daphnia pulicara*	Pokey 5 kb
ABS18391	*Helicoverpa armigera*	transposase
BAD07480	*Bombyx mori*	Yabusame-w
BAD11135	*Bombyx mori*	Yabusame-1
BAD11136	*Bombyx mori*	Yabusame-2
BAF82017	*Xenopus laevis*	Kobuta
BAF82019	*Xenopus tropicalis*	Uribo-1
BAF82020	*Xenopus laevis*	Uribo-1
BAF82021	*Xenopus borealis*	Uribo-1
BAF82022	*Xenopus tropicalis*	Uribo-2
CAF99963	*Tetraodon nigroviridis*	Unnamed Product
CAI18712	*Homo sapiens*	PGBD-2
NP_689808	*Homo sapiens*	PGBD-4
NP_741958	*Mus musculus*	PGBD-5
Q8N328	*Homo sapiens*	PGBD-3
Q8N414	*Homo sapiens*	PGBD-5
Q96JS3	*Homo sapiens*	PGBD-1
XP_001030237	*Tetrahymena thermophila*	Predicted protein
XP_001541487	*Ajellomyces capsulatus*	Predicted protein
XP_001599370	*Nasonia vitripennis*	Predicted protein
XP_312615	*Anopheles Gambiae*	PGBD
XP_590785	*Bos taurus*	Predicted protein
XP_699416	*Danio rero*	Predicted protein
XP_797885	*Strongylocentrotus purpuratus*	Predicted protein

GenBank accession number, taxon, common name, and protein name of the proteins used in the ClustalW alignment.

**Figure 1:**
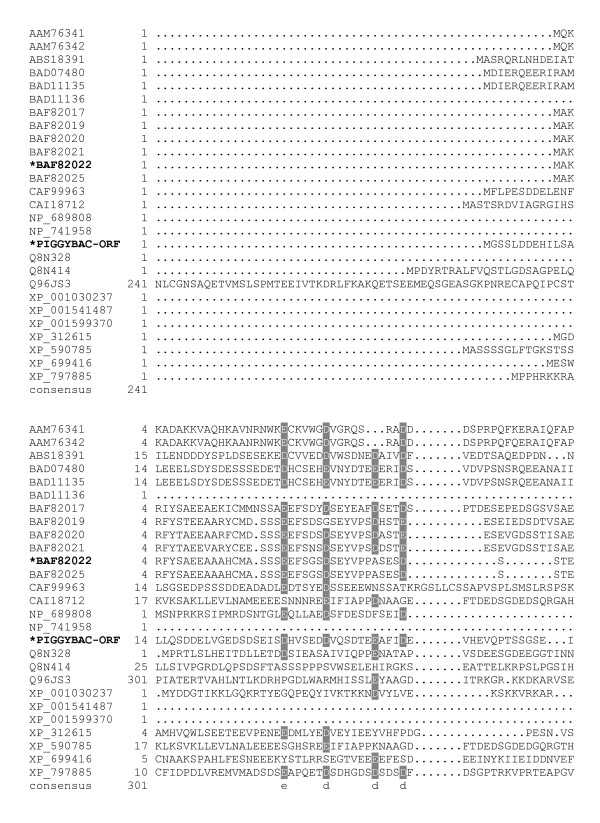
**Alignment of *piggyBac*-related proteins**. A BoxShade server alignment of the proteins listed in table 1. Residues aligning with *piggyBac *position 1–63 are shown. Only two *piggyBac *family proteins have been shown to catalyze transposition, these are indicated by bold face type and asterisks.

**Figure 2:**
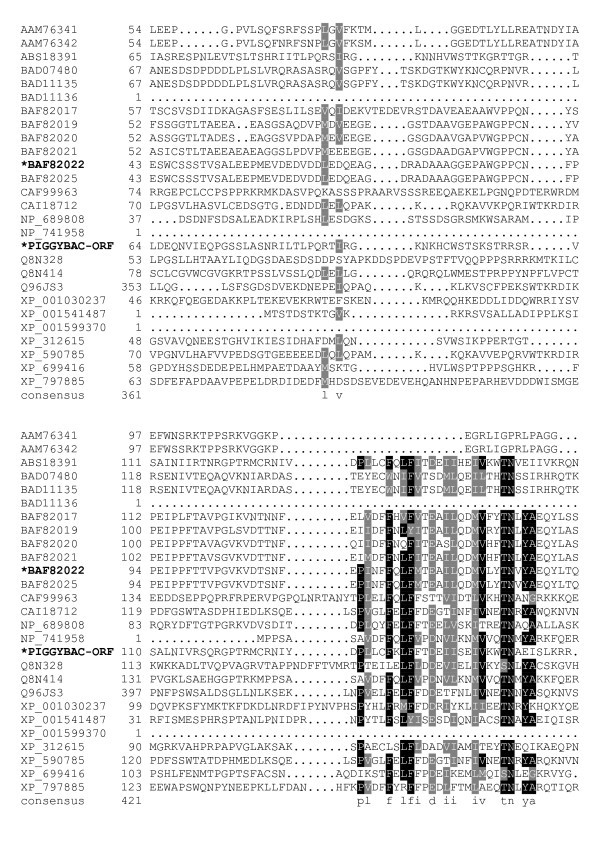
**Alignment of *piggyBac*-related proteins**. A BoxShade server alignment of the proteins listed in table 1. Residues aligning with *piggyBac *position 64–160 are shown. Only two *piggyBac *family proteins have been shown to catalyze transposition, these are indicated by bold face type and asterisks.

**Figure 3:**
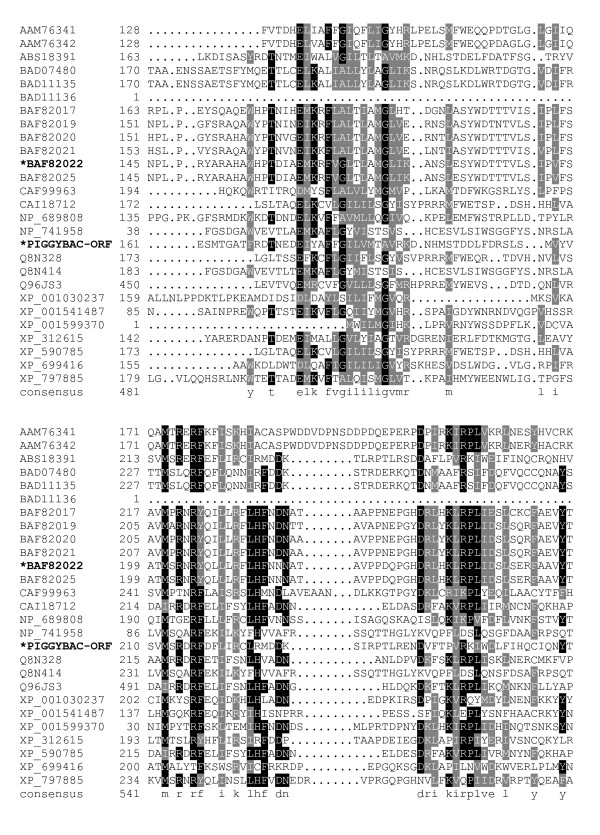
**Alignment of *piggyBac*-related proteins**. A BoxShade server alignment of the proteins listed in table 1. Residues aligning with *piggyBac *position 161–260 are shown. Only two *piggyBac *family proteins have been shown to catalyze transposition, these are indicated by bold face type and asterisks.

**Figure 4:**
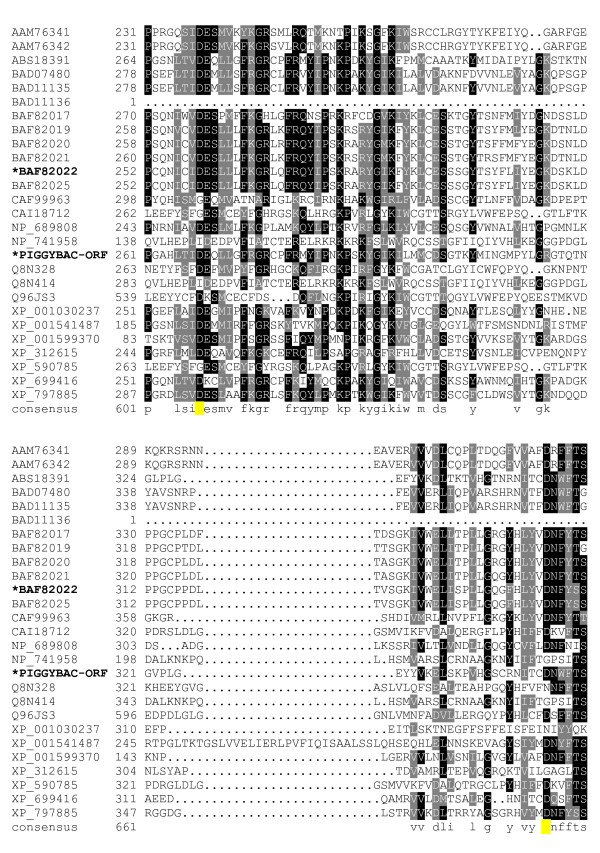
**Alignment of *piggyBac*-related proteins**. A BoxShade server alignment of the proteins listed in table 1. Residues aligning with *piggyBac *position 261–351 are shown. Only two *piggyBac *family proteins have been shown to catalyze transposition, these are indicated by bold face type and asterisks. The aspartate residues mutated in this study are highlighted in yellow.

**Figure 5:**
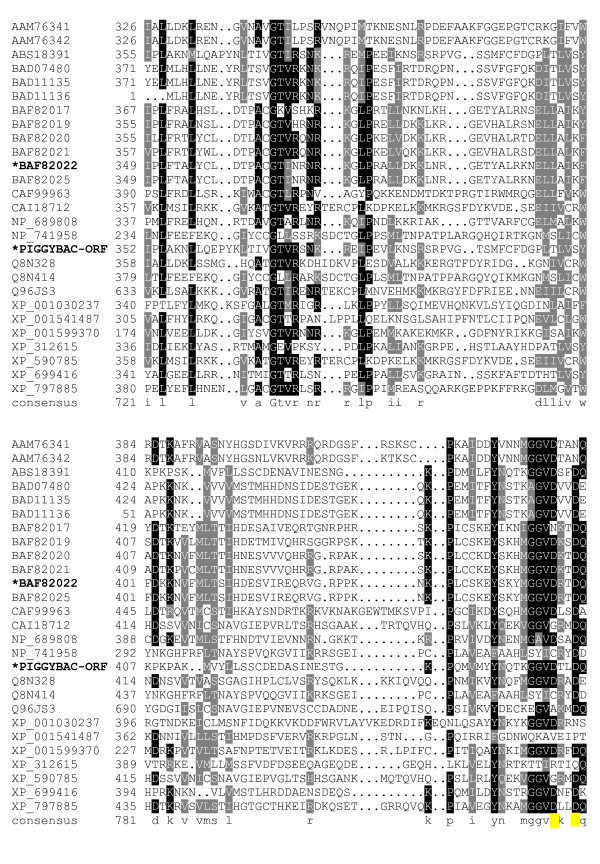
**Alignment of *piggyBac*-related proteins**. A BoxShade server alignment of the proteins listed in table 1. Residues aligning with *piggyBac *position 352–451 are shown. Only two *piggyBac *family proteins have been shown to catalyze transposition, these are indicated by bold face type and asterisks. The aspartate residues mutated in this study are highlighted in yellow.

**Figure 6:**
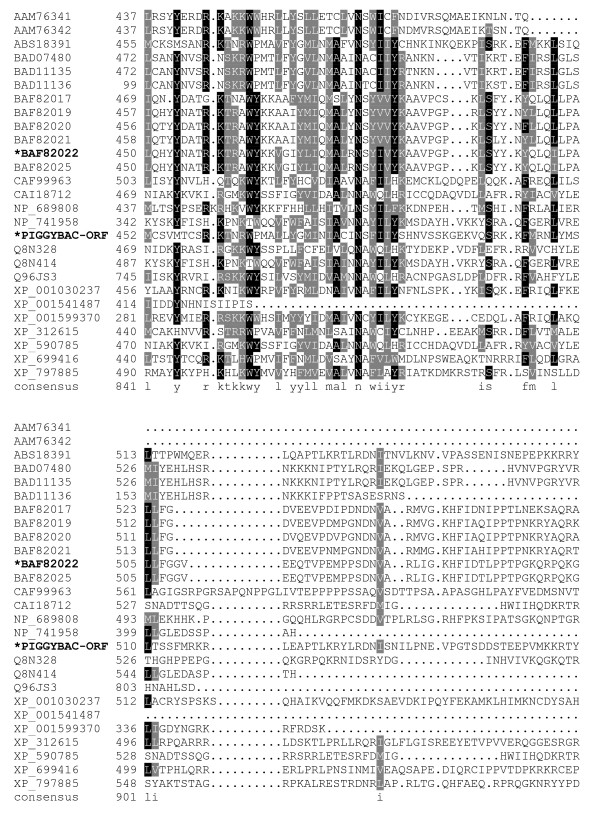
**Alignment of *piggyBac*-related proteins**. A BoxShade server alignment of the proteins listed in table 1. Residues aligning with *piggyBac *position 452–558 are shown. Only two *piggyBac *family proteins have been shown to catalyze transposition, these are indicated by bold face type and asterisks.

**Figure 7:**
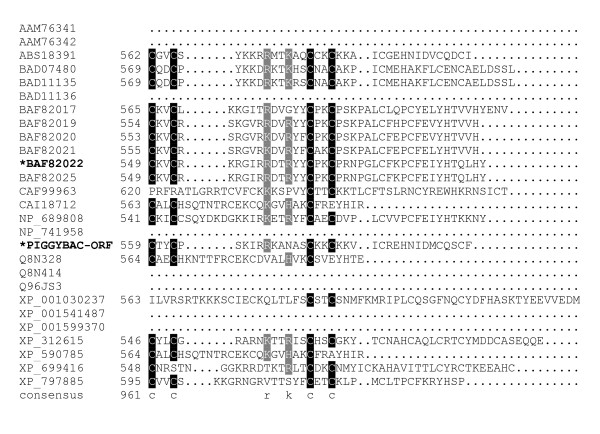
**Alignment of *piggyBac*-related proteins**. A BoxShade server alignment of the proteins listed in table 1. Residues aligning with *piggyBac *position 559 through the c-terminus are shown. Only two *piggyBac *family proteins have been shown to catalyze transposition, these are indicated by bold face type and asterisks.

The sequence alignments (Fig. 1, 2, 3, 4, 5, 6, 7) immediately revealed several highly conserved features. Starting at the N-terminus of the transposase alignment, we noticed four conserved acidic residues: D32, D38, E45, and D49 (Fig. 1). The presence of either an aspartate or a glutamate at these positions does not seem to matter even within families; the acidic charge is the only conserved feature we can distinguish.

The core of the transposase possesses clusters of conserved amino acids with very well defined borders for each conserved stretch. The first conserved cluster we identified begins shortly downstream of a conserved proline, P131, and may mark the beginning of a functional domain in *piggyBac *transposase family members. The next clusters of conservation lie roughly from P131 – N152, E175 – K190, and V207 – D228 (Fig. 2, 3). The region between D239 – Y259, while not particularly conserved in *piggyBac *itself, is conserved in other members of the family. Additionally, the next proline from this cluster, P261, starts another highly conserved region which includes the first member of the proposed 'DDD' triad, D268 (Fig. 4).

The next group of conserved residues contains the second aspartate, D346 and an absolutely conserved glycine at position 369. G369 marks the start of the well conserved motif 'GTVRxNKRxIP' (Fig. 5). This motif ends with P379, possibly delineating the end of a functional domain. This motif is not present in its entirety in Uribo-2. However, the analogous sequence of Uribo-2 'GTINRNRKxLP' does preserve similar features.

Downstream from this region is another conserved group that starts with a highly conserved proline at position 433. P433 is only a short distance from D447 and D450, the last two aspartates tested in our study, and is the start of another motif: 'PxxxxxxYxxxxGGVDxxD.' The aspartates in this motif are D447 and D450, respectively (Fig. 5, 6).

A cysteine-rich domain overlapping with the *piggyBac *nuclear localization signal (NLS) is positioned at the C-terminus of *piggyBac*. The spacing between these cysteines is well conserved among *piggyBac *family members when present, even though the intervening amino acids are not. Additional cysteines also lie within this region, conforming to a novel zinc-finger domain in *piggyBac *[52,53]. A basic amino charge at R568 is also well conserved (Fig. 7).

### Excision assay

**Figure 8:**
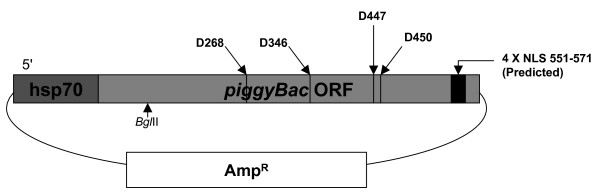
**Diagram of *piggyBac *ORF and genotypes used in this study**. A diagrammatic representation of the *piggyBac *transposase mutant genotypes used in this study. The entire *piggyBac *ORF is displayed with the aspartates noted with their corresponding residue number. 4 × NLS represents 4 PSORT predicted nuclear localization patterns as described in discussion.

**Figure 9:**
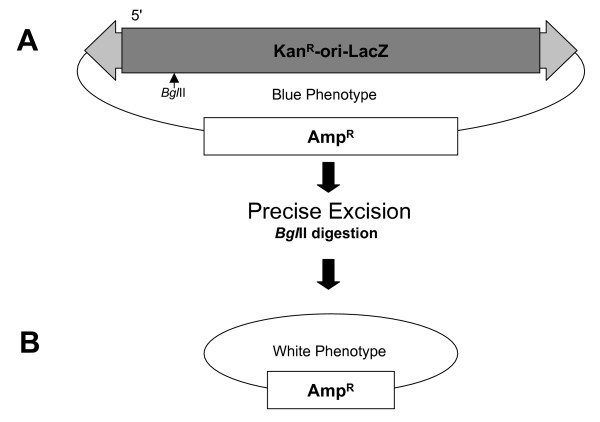
**Schematic of the REN colony screen**. (A) The donor plasmid pBKOα is co-transfected with one of the *piggyBac *expression plasmids (fig. 8), transformed into *E. coli *and plated. (B) Precise excision of the *piggyBac *cassette removes the lacZ open reading frame. Digestion with *Bgl*II removes any excess *piggyBac *expression plasmids and donor plasmids which have not undergone excision. Excision events are scored as white colonies. Plating a portion of undigested plasmids and scoring blue colonies yields the total number of potential donor plasmids.

The excision activity of a nonautonomous *piggyBac *element from the donor plasmids pBKOα [32] was assessed for 8 mutant transposases relative to the wild-type transposase (Fig. 8; Fig. 9). Plasmid DNA was extracted from transfected cell cultures and transformed into *E. coli *cells. As with previous assays [32], the number of non-excised plasmids was reduced by restriction digestion of a site unique to the donor element. The large number of colonies precluded analysis of every transformant, but a representative cross section was chosen for analysis by restriction enzyme digestion. Positive events were further screened by sequence analysis. Only events that showed precise excision were tallied.

**Figure 10:**
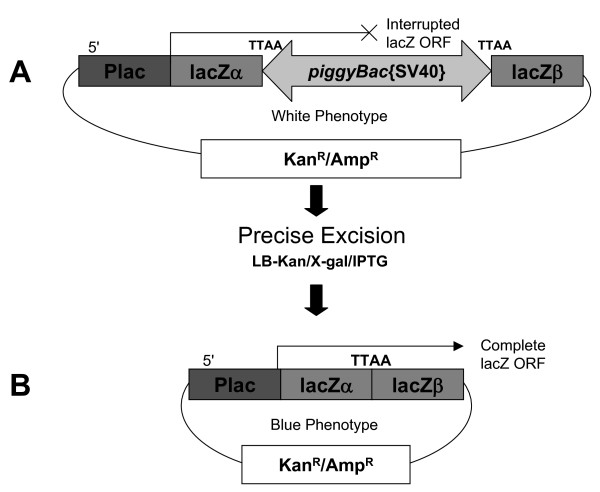
**Schematic of the blue/white colony excision assay**. (A) The donor plasmid pCR2.1-*piggyBac*{SV40} is co-transfected with one of the *piggyBac *expression plasmids (fig. 8) transformed into *E. coli *and plated. (B) Precise excision of the *piggyBac*{SV40} cassette restores the original lacZ open reading frame at the original 'TTAA' insertion site. By plating on kanamycin media containing IPTG and X-gal, only *E. coli *containing donor plasmids will develop into colonies. Colonies which are blue represent excision events, while the number of blue and white colonies represents the total number of potential donor plasmids.

**Figure 11:**
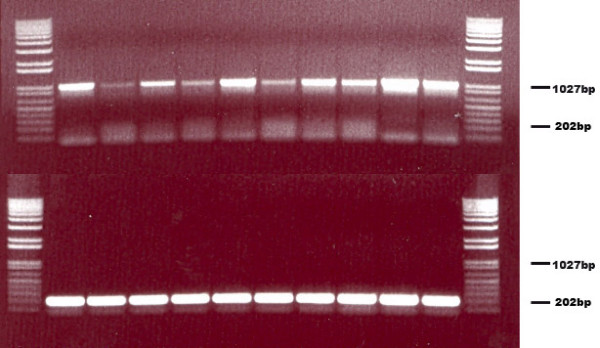
**PCR analysis of donor plasmid sizes from the blue/white colony screen**. PCR of 10 white (negative, top row) colonies and 10 blue (positive, bottom row) colonies, randomly chosen, from the blue/white colony screen is shown on a .9% TAE agarose gel stained with ethidium bromide. The amplicon crosses the entire donor fragment. Expected band size for the pre-excision plasmid is 1027 bp and 202 bp for the post-excision donor plasmid amplicon.

In order to obtain the largest amount of data and to ascertain if differences in the donor plasmid had any effect on excision efficiency, a second assay employing a blue/white colony screen was developed using the Topo-TA vector backbone, pCR2.1-Topo (Invitrogen, Carlsbad, CA) to make the donor plasmid pCR2.1-*piggyBac*{SV40}. Precise excision events would restore the 'TTAA' sequence of the region between the two lacZ coding sequence fragments, allowing for the expression of a fully active β-Gal protein. This assay allowed rapid screening of large numbers of colonies and identified positive excision events (Fig. 10). A simple PCR of blue colonies confirmed that excision had occurred (Fig. 11). Subsequent sequencing of some of these blue colonies validated these findings.

Our mutational analysis demonstrates that all members of the proposed triad, D268, D346, and D447, are required for efficient *piggyBac *catalyzed excision in a cellular environment. The additional amino acid targeted for mutational analysis, D450, has a primary requirement for a negative charge, since the asparagine replacement significantly inhibited excision, while a glutamate replacement yielded moderate excision, although not to the degree of the wild-type transposase.

**Figure 12:**
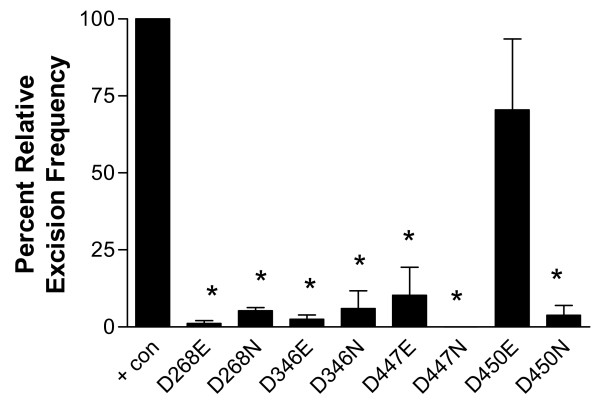
**Relative frequency of excision obtained with the REN colony screen**. Positive control, wild-type *piggyBac*, is set to 100. Data are expressed as a mean of three replicates +/- standard error bars. ANOVA test was performed using GraphPad Prism 3.0 software. Means were considered statistically significant when p-values less than 0.05 were obtained with the Dunnett's post-test. Statistical significance of difference with regards to positive control is indicated on all data figures as asterisks above bars. (p < 0.05)

**Figure 13:**
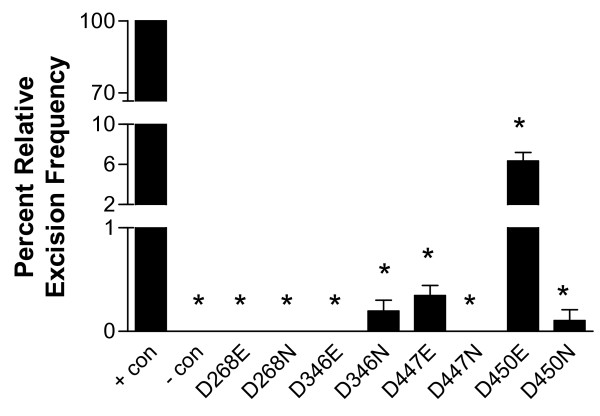
**Relative frequency of excision obtained with the Blue/White colony screen**. Positive control, wild-type *piggyBac*, is set to 100. Data are expressed as a mean of three replicates +/- standard error bars. ANOVA test was performed using GraphPad Prism 3.0 software. Means were considered statistically significant when p-values less than 0.05 were obtained with the Dunnett's post-test. Statistical significance of difference with regards to positive control is indicated on all data figures as asterisks above bars. (p < 0.05)

Even though the trends remained the same, a difference in overall excision efficiency between donor plasmids was also demonstrated. The pBKOα plasmid donor element was able to facilitate some level of excision for all mutant transposases except D447N (Fig. 12). In contrast, despite the larger number of colonies screened, pCR2.1-*piggyBac*{SV40} excision failed to yield events for D268E, D268N, D346E, and D447N (Fig. 13). When precise excision was catalyzed, all frequencies were less than that of the pBKOα counterpart when the same helper plasmid was utilized (Table 2). Incomplete digestion of the helper plasmid in the REN screen excision assay prior to plating (see: methods) may have been a contributing factor, leading to artifact colonies which would inflate the actual number of excised colonies. However, excision efficiency may be influenced by other factors, such as donor fragment size or the physical properties of the DNA at the excision sites. The size of the excision fragments of the two donor plasmids vary considerably: 6218 bp for pBKOα and 825 bp for pCR2.1-*piggyBac*{SV40}.

**Table 2:** Excision frequencies for donor plasmids

	Blue-White		REN Screen	
		
Mutant	Excision Frequency	+/- SE	Excision Frequency	+/- SE
D268E	0.00E+00	0.00E+00	9.96E-03	3.73E-03
D268N	0.00E+00	0.00E+00	8.03E-05	4.73E-05
D346E	0.00E+00	0.00E+00	8.97E-05	4.90E-05
D346N	2.97E-05	1.49E-05	3.62E-04	2.02E-04
D447E	4.44E-05	8.54E-06	2.40E-04	1.84E-04
D447N	0.00E+00	0.00E+00	3.99E-04	2.93E-04
D450E	8.80E-04	1.36E-04	0.00E+00	0.00E+00
D450N	1.38E-05	1.38E-05	1.25E-03	0.00E+00
+ctrl	1.40E-02	2.12E-03	3.15E-03	8.86E-04
-ctrl	0.00E+00	0.00E+00	00E+00	0.00E+00

Values obtained for the frequency of excision using the two different donor plasmids with each of the mutant transposases. These numbers are normalized to a positive control frequency of 100%.

### Western blot

**Figure 14:**
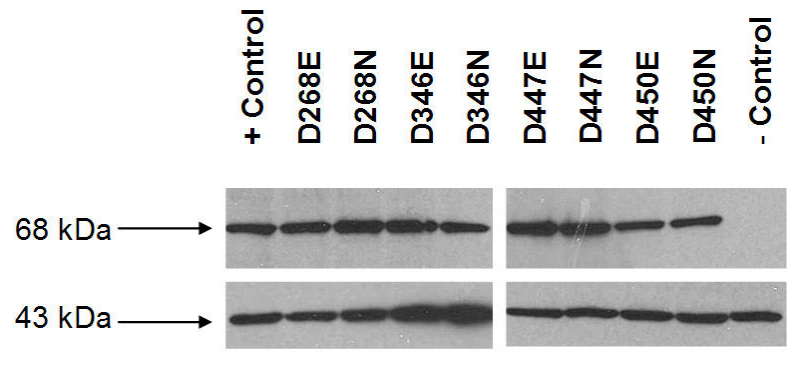
**Western blot of *piggyBac* mutant transposases**. A western blot was performed on each *piggyBac* mutant with 125 μg total cell lysate per lane. The top row was probed with anti-*piggyBac *antibody and indicates the presence of the *piggyBac *transposase at 68 kDa in each mutant, the positive control, phsp-pBac, and a lack of transposase in the negative control. The bottom row was probed for actin, a 43 kDa protein, using anti-actin I-19 and shows equal loading in all lanes.

A western blot was performed to ensure that full length *piggyBac *transposase was being produced from the expression vectors. This was done to verify that any mutant expression vectors unable to drive excision activity were a result of the mutations in the transposase and not for a lack of expression through faulty vectors. All mutant vectors expressed equivalent amounts of protein of approximately 68 kDa to which a polyclonal *piggyBac *antibody was able to bind. All lanes probed positive for actin, used as a loading control (Fig. 14).

## Discussion

Sarkar et al. suggested the presence of a 'DDD' motif for *piggyBac*, without empirical evidence of the association of the motif with mobility. Their proposal was based on two weak matches returned when two *piggyBac *related proteins, but not *piggyBac *itself, were used as a query in an NCBI Conserved Domain Search. These query hits were for the Pfam domain pfam01609 [48]. The conserved D268 and D346 were thus matched to the bacterial *IS*4/5 transposase family DDE motif based on their degree of conservation in the transposase core and the presence of a glutamate immediately following D268, and an asparagine following D346. No reason for choosing D447 was cited, but both D447 and D450 are part of a highly conserved motif discussed below.

Bigot and colleagues [54] used a similar approach of transposase alignments to propose the existence of a 'DDE' motif in *hAT *elements, with the second aspartate replaced by a serine in *Ac*, *hobo*, and *Hermes*. They aligned members of the *Tc1-mariner *superfamily with members of the *hAT *family and identified conserved and similar residues common between both groups. The 'DSE' speculation has since been empirically disproven [55]. However, of this 'DSE' triad, both D402 and E572 (with respect to *Hermes*) were essential for transposition while alteration of S535 to either an alanine or an aspartate had no statistically significant effect on transposition efficiency. This, at least, demonstrated the necessity for D402 and E572, but did not prove if either residue was part of an essential triad.

Starting at the N-terminus of *piggyBac *there are four acidic amino acids: D32, D38, E45, and D49 that are present in most of the aligned proteins (Fig. 1). While charges are conserved at these positions, the residues themselves do not seem to have any requirement as to whether they are an aspartate or a glutamate. Interestingly, this interchangeability is particularly variable at these positions, even within closely related proteins, such as the *piggyBac *related proteins in three different species of *Xenopus*, and among the human *piggyBac *derived proteins (PGBD). Examination of *piggyBac *related transposons in *Xenopus *identified three elements, Uribo-1, Uribo-2, and Kobuta. Xtr-Uribo2_PCR_Iv1b proved to be a functional mobile element complete with transposase able to catalyze movement in GP293 cells [49]. Kobuta, however, lacks excision activity. Xtr-Uribo2_PCR_Iv1b possesses a glutamate at *piggyBac*'s D32 and does not have a match for E45, even though other inactive *Xenopus *putative transposases, Uribo-1 and Kobuta, do have matches for E45. Additionally, these N-terminal acidic residues are spaced so closely together that they are unlikely to be the DDD/DDE triad in our opinion. Interestingly, both Uribo proteins contained analogs to D268, D346, and D447, while the inactive Kobuta protein contained only D346 as a rule with two divergent Kobuta examples also having the D268 residue. All *Xenopus *proteins also harbor the highly conserved D450 residue.

Distinct clusters of conserved amino acids are present through the rest of the *piggyBac *family starting at P131 (Fig. 2). The fact that the first constellation of conservation begins with a proline is worth noting, as proline is known to disrupt the periodic structure of α-helices and β-sheets, often demarcating the protein from one functional domain to the next [56]. Together with the adjacent region of conservation, we speculate that the area just downstream of P131 is most likely a functional domain in the transposase. A conserved domain search returns an extremely weak (e = .44) match to pfam02388, *Staphylococcus *proteins involved in formation of the peptidoglycan layer, a coincidence in our opinion.

The *piggyBac *family analog to K246 is also a highly conserved proline, but as this amino acid is not present in *piggyBac *it is not required for a functional transposase (Fig. 3). However, P261, while less conserved than the K246 proline analogs, is present in both *piggyBac *and Uribo-2. It also lays just N-terminal of the very well conserved residues D268, the first member of the 'DDD' triad, and E269.

The next cluster of conserved amino acids includes D346, the second member of the proposed 'DDD' triad (Fig. 4). Also in this region lies the only residue in the alignment that is absolutely conserved is G369 (Fig. 5). This glycine is the start of a nearly universally conserved motif among *piggyBac *related proteins: 'GTVRxNKRxIP.' While R372 is limited to arginine, the other two basic amino acids, K375 and R376, seem to be conserved only in charge, as some proteins use either arginine or lysine at these positions. When a basic amino acid occupies sites analogous to both position 375 and 376 in a protein, it is always one of each and never the same residue, except for *Strongylocentrotus purpuratus *which utilizes arginines in both locations. I378 is also another residue conserved only in properties, in this case hydrophobicity. Methionine, leucine, and isoleucine each are employed at this site with no clear pattern as to which is used outside of immediately related members. Finally, P379 does not seem to indicate the start of any highly conserved clusters, but it does lie at the end of a conserved cluster starting at approximately E331, possibly ending a functional domain in *piggyBac*. Of note, this motif is not present in its entirety in the functional transposase, Uribo-2. However, the analogous sequence of Uribo-2 'GTINRNRKxLP' does preserve similar features.

The next highly conserved proline, P433, is located only a short distance from P379, just upstream of the third proposed aspartate, D447, and another highly conserved aspartate at D450 (Fig. 5). This proline delineates the start of one of the most highly conserved motifs we found in our alignment: 'PxxxxxxYxxxxGGVDxxD' which contains both D447 and D450. This motif is present in both *piggyBac *and Uribo-2 as well as most other members of our analysis. Among members where this motif is loosely present, G444 is replaced by an alanine in the silkworm Yabusame proteins, and G445 is replaced with an alanine in the human PGBD-4. P433 could mark the start of another required functional domain in *piggyBac*, and for this reason we decided to mutate both D447 and D450. In the more divergent proteins, mouse PGBD-5, human PGBD-5, human PGBD-1, *Anopheles gambiae *PGDB protein XP_312615, and *Ajellomyces capsulatus *predicted protein XP_001599370, this motif is not fully present.

The C-terminus of most *piggyBac *related proteins contains a cysteine-rich motif: C559, C562, C574, C577, and C582 (Fig. 7). The spacing between these residues is somewhat conserved even though the intervening residues are not. In *piggyBac*, this region is also a novel match for a RING-finger motif [52], a type of Zinc-finger which has been implicated in protein-protein interactions [53]. The high degree of conservation in this region suggests a selective pressure for the presence and spacing of these cysteines. Recently, Mitra and colleagues [34] have suggested this Zinc-finger could in fact be a PHD finger, implicated in heterochromatin interactions. The ability of their purified transposase to function in an *in vitro *environment on free, unwound DNA despite the removal of this C-terminal domain is consistent with this hypothesis. The occurrence of a basic charged residue at R569 is also a commonality.

In *piggyBac*, we have demonstrated that this area contains a functional NLS (aa 551–571) (in press). In fact, this region is so rich with basic amino acids, that 4 PSORTII [57] predicted NLS matches exist in this short region: 2 monopartite at positions 551 and 563, and 2 bipartite signals at positions 554 and 555, respectively. Interestingly, this NLS cluster is not present in the functional Uribo-2 transposase, however a PSORT analysis of Uribo-2 shows it harbors a NLS matching a monopartite pattern beginning at P276, with respect to the Uribo-2 sequence.

The excision assays reveal that substitution of D268, D346, D447, or D450 with either an asparagine or a glutamate impairs the function of the transposase from the donor plasmid pCR2.1-*piggyBac*{SV40}. In contrast, while the substitution D450E significantly impaired excision from pCR2.1-*piggyBac*{SV40}, it did not appear to have a statistically significant effect with regards to pBKOα. The reasons for this apparent difference reflect level of error inherent in the protocols between the two assays. For the REN colony screen, a randomly chosen representative cross section is cultured and digested from the colonies available. This method is prone to a larger amount of error as fewer colonies are screened for excision events. However, the blue/white colony screen permits the analysis of all the colonies on a plate for excision events. This provides a much larger population of donor plasmids to be analyzed and reduces the standard error.

These four residues mutated in our study are far from the only conserved acidic amino acids in the *piggyBac *family alignment: the short cluster at the amino terminus previously discussed, as well as D141, E175, D249, and D300 are all conserved to a degree, and may be involved in transposition. Each of the mutations tested severely reduced or completely shut down excision activity of the transposase. Previous studies of acidic amino acids in *Hermes *support the idea that essential amino acids are not limited to just the three members of a proposed triad, as alterations to D180, D248, D402, and E572 in the *Hermes *transposase all affect transpositional activity [55,58].

Here, we test only the excision step of the entire transposition reaction in a cell culture system. There are other possibilities that cannot be ruled out by our data, including the necessity of these residues in forming a functional secondary structure of the transposase. We cannot say for certain which specific parts of the excision process these mutations hinder. It is entirely possible that they are required for DNA binding or possibly the recruitment of auxiliary host factors. However, the reports of Mitra *et al. *[34] examining *piggyBac *in function an *in vitro *system suggests that the transposase alone may carry out all steps of transposition without any additional requirement for host factors. Substitution of D268, D346, and D447 with alanine does not inhibit specific binding of the transposase to the terminal repeats of *piggyBac *[34], but each of these mutations abrogates all steps of transposition, including 3' OH nicking and hairpin formation – both integral steps for excision. Furthermore, unlike wild type *piggyBac*, these mutants are defective for target joining when synapsed with an artificial excision fragment. Testing these mutants in a yeast system also demonstrated they were functionally defective [34]. The lack of catalytic activity in our D268, D346, and D447 mutant assays confirms the relevance of the observation of Mitra *et al. *to *piggyBac *movement in insect cell systems.

In contrast, Mitra *et al. *demonstrate that D450A is still catalytically active in an *in vitro *environment, while our results show a definite interfering effect. One explanation for this discrepancy is that D450 is likely not a part of the metal interaction motif, but may be involved in the proper folding of the protein with limited tolerances for certain amino acids. Certainly, our findings support that D450 is the least critical of the four aspartates we tested, but is still necessary to a degree.

Just because an amino acid is acidic and required for transposition does not automatically make it a member of a divalent metal interaction motif, nor does particular mutant interfere with all steps of transposition. An example of this can be found in a mutational study of the V(D)J recombination initiator, RAG1. In this experiment, a great number of acidic amino acids were mutated, some of which impaired cleavage activity. One of these mutations, E811Q, was deficient for DNA binding activity, a loss-of-function that could indirectly lead to decreased cleavage activity. This study also defined two classes of mutants: class I which retained a measure of cleavage activity, and class II which yielded no detectable cleavage products, even though they retained DNA binding activity [42]. It is possible that alteration of D450 in *piggyBac *may be analogous to such a class I mutant.

Another example of a mutant transposase deficient for specific steps of transposition would be the D248A mutant of *Hermes*, which was deficient for DSB activity. Upon further investigation, this mutant appeared to only be deficient in the first 5' nicking step of excision. When supplied with a pre-nicked substrate, it was able to facilitate hairpin formation and a measure of target joining in the presence of Mn^2+^, the second step of DSB and the final step of transposition, respectively [58]. These examples illustrate that a number of steps exist in which a mutant could interfere with transposition, thereby decreasing the formation of the end product.

Iron-induced hydroxyl radical protein cleavage is one direct test for metal binding activity [59]. This assay takes advantage of the ability of Fe^2+^, in the presence of ascorbate or H_2_O_2_, to generate hydroxyl radicals. If the protein in question has the ability to bind Fe^2+^, then hydroxyl radicals will be generated at the sites where the ion is located. These radicals subsequently cleave the peptide backbone of the protein which bound the Fe^2+^, yielding cleavage products with sizes consistent with the location of the metal binding pockets. Regarding recombinases, this approach was used to narrow down the location of the metal binding residues of RAG1 prior to the generation of site-specific mutants [43].

Another common test for metal interaction is the replacement of either the aspartate or the glutamate with a cysteine [60]. This test is based on the ability of glutamate and aspartate metal ligand residues to supply a carboxyl group for metal interaction. These amino acids use the oxygen on their R-chain as the interacting atom via O-Mg^2+ ^or O-Mn^2+ ^formation. However, interactions with Mn^2+^, but not Mg^2+^, are also able to occur using a cysteine via a S-Mn^2+ ^bond [61]. Such an interaction can be analogous to the metal binding activities of glutamate and aspartate. For example, substitutions D180C, D248A, and D248C in *Hermes *all showed the ability to catalyze hairpin formation of substrate DNA in the presence of Mn^2+ ^during the excision step of transposition, but only D180C and D248A were able to complete the joining of the transpososome to target DNA. E572A and E572C left *Hermes *unable to facilitate any step of the transposition reaction [58].

Finally, much work has been done with X-ray crystallography on integrases, resolvases, and transposases at different stages of catalysis, including multimers, free floating proteins, and enzymes complexed with their target DNA. Direct resolution of these structures has been invaluable to the understanding of structure-function relationships. Indeed, the various studies have found structural similarities in the active sites of the recombinase family and nucleases, including the DDD/DDE metal binding motif for both one and two metal binding proteins [62-69]. In light of what we and others have found [34] it would not be surprising to find *piggyBac *shared many features common to the recombinase family.

## Conclusion

The *piggyBac *family alignment revealed a number of interesting features in the transposase. Regions of high conservation in the catalytic core were sometimes demarcated by proline residues, possibility separating functional domains. Furthermore, we found four conserved acidic residues at the N-terminus of the transposase, and four more throughout the rest of the transposase. A cysteine-rich region with somewhat conserved spacing exists at the C-terminus and is a novel match for the RING finger protein-interaction motif. Our data indicate which of the four tested residues are essential for transposase-mediated excision from a donor plasmid in *Drosophila *cultured cells. Our donor plasmids yielded similar results, however a charge-preserving substitution from aspartate to glutamate was at least moderately tolerated at positions D450. The existence of an acidic charge was necessary at all four positions tested for both of our donor plasmids with a specific requirement for an aspartate at D268, D346, and D447. We conclude that the four amino acids tested are indeed vital for efficient excision of the *piggyBac *element from a donor plasmid in cell culture.

## Methods

### Sequence retrieval and alignment

The predicted *piggyBac *translated protein was used to scan GenBank [50] in a protein-BLAST search of the non-redundant database. Hits that were not from *Trichoplusia ni *were chosen for a multiple sequence alignment in ClustalW [70]. The resulting .msf alignment was input into the BoxShade server in order to more easily identify the conserved regions.

### Plasmid Construction

pXL-BacII-SV40 was constructed by amplifying the SV40 terminator sequence from pMT/V5-HisA (Invitrogen, Carlsbad, CA) with the primers: Sense: 5' TGTGCGGCCGCAGTCTAGAAAAGGATCCTAGATCATAATCAGCCATACCACATTTGTAGAGG 3' and antisense: 5' TGGGAGCTCATAAGCCGTATCGATAAGCTTTAAGATACATTGATG 3' with *Pfx *high-fidelity polymerase (Invitrogen) according to manufacturer protocol. The PCR reaction was separated on .9% agarose gel stained with 10 μg/ml ethidium bromide and the band corresponding to the SV40 fragment was isolated with the Wizard SV Gel Purification System (Invitrogen). The fragment was then cut with *Bam*HI (New England Biolabs (NEB), Ipswitch, MA) and *Cla*I (NEB) and ligated into the vector pXL-BacII [71] at the same sites to form pXL-BacII-SV40. The plasmid was sequenced to verify accuracy.

To create pCR2.1-*piggyBac*{SV40}, the *piggyBac*{SV40} fragment was amplified from pXL-BacII-SV40 with the primers: Sense: 5' AAACCCAAAGGTACCGAGCTCGGATCCACTAGTAACGGCCGCCAGTGTGCTGGAATTCGGGG**TT****AA**CCCTAGAAAGATAATCATATTGTGACG 3' and Antisense: 5' CCCAAACCCGCGGCCGCCAGTGTGATGGATATCTGCAGAATTCGGGG**TT****AA**CCCTAGAAAGATAGTCTGCGTAAAATTG 3' using *Pfx *high-fidelity polymerase. The 'TTAA' sites delineating the ends of the *piggyBac *donor fragment are in bold while the sequences remaining behind after precise excision are underlined. The fragment corresponding to *piggyBac*{SV40} was band-isolated as described above and cut with the restriction enzymes *Acc*65I (NEB) and *Not*I (NEB) This fragment was then ligated into the vector pCR2.1-Topo (Invitrogen) at the same sites. The plasmid was sequenced to verify accuracy.

The various mutants of the *piggyBac *open reading frame were prepared by Bio Basic Inc. (Biobasic Inc., Ontario, Canada) in the vector backbone phsp-pBac [12] according to the specifications listed in Table 1.

### Blue/White colony screen

The blue/white colony screen was developed to enable us to determine, by visual inspection, which colonies had undergone precise excision. The *piggyBac*{SV40} cassette was inserted at a 'TTAA' site in the pCR2.1 backbone, in the midst of the lacZ ORF. The *piggyBac *expression plasmid and the pCR2.1-*piggyBac*{SV40} are co-transfected into *D. melanogaster *Schneider2 (S2) cells and incubated. The plasmids are recovered by Hirt extraction and transformed into *E. coli*. In the presence of the cassette, the lacZ ORF is interrupted, yielding a white colony phenotype on kanamycin containing media with IPTG and 5-bromo-4-chloro-3-indolyl-b-D-galactoside (X-gal). However, when precise *piggyBac*-catalyzed excision occurs in this vector, the lacZ ORF is restored and the subsequent *E. coli *colony will possess a blue phenotype (Fig. 10). Plating on kanamycin containing media ensures that only donor plasmids will develop into colonies, and helper expression plasmids will not lead to false white colonies. By comparing the number of blue colonies to the number of total (blue+white) colonies, we can extrapolate the excision frequency. That is, the number of excision events occurring out of the total number of potential excision events.

10^6 ^S2 cells were allowed to adhere to the bottom of a 9.6 cm^2 ^cell culture well and were transfected with .4 μg of phsp-pBac helper plasmid and .4 μg of pCR2.1-*piggyBac*{SV40} donor plasmid with Transfectin liposomal transfection reagent (Bio-Rad Laboratories, Hercules, CA) according to manufacturer protocol in minimal S2 media (Gibco, Carlsbad, CA). The cell/transfection media mixture was allowed to sit at room temperature overnight. The next morning, the transfection media was aspirated from the cells and replaced with complete S2 media (Minimal S2 supplemented with 10% FBS, .1 mg/mL streptomycin, and .25 μg/mL amphotericin). The cells were heat-shocked to induce phsp-pBac promoter activation at 37°C for 60 minutes. The cells were then moved to a 28°C incubator for 48 hours prior to harvesting.

Plasmid DNA was isolated from the S2 cells according to the standard Hirt LMW DNA extraction protocol [72]. Additional steps taken were as follows: Following the centrifugation of the lysate, the DNA-containing supernatant was removed, extracted once in phenol:chloroform:isoamyl alcohol (25:24:1) and washed once with chloroform:isoamyl alcohol (24:1). DNA was precipitated with 2 volumes 100% ethanol and 1/10 volume 3 M sodium acetate (pH 4.5) and stored at -20°C overnight. DNA was pelleted by centrifugation at 14,000 rpm at 4°C and the supernatant aspirated. The pellet was washed 3 times with 70% ethanol to remove the excess salt from the Hirt extraction buffers, dried, and resuspended in 40 μl of nuclease-free water. 1 μl of the Hirt extraction DNA was mixed in 21 μl nuclease-free water and transformed by electroporation into 3 μl *E coli*. DH10B cells (Invitrogen) in a 1 mm gap electroporation cuvette according to manufacturer protocol. 150 μl of S.O.C. medium was pipetted directly into the electroporation cuvette and mixed well by pipetting. Cells were allowed to recover at 37°C for 30 minutes prior to plating. 10 μl and 100 μl portions of this cell-media mixture were spread onto LB-Kanamycin plates prepared with 4 μl 100 μg/μl IPTG and 70 μl 20 mg/ml X-gal. The plates were incubated overnight at 37°C and scored by counting the number of blue colonies on the plate spread with 100 μl cell-media mixture and the total number of colonies on the plate spread with 10 μl of the cell-media mixture.

### PCR verification of excision events

A portion of the blue colonies were screened for excision events to verify accuracy of the test. Briefly, a 20 μl total volume PCR was prepared with a final concentration of 50 mM KCl, 10 mM Tris pH 8.3, 1.5 mM MgCl_2_, 5 units of *Taq *polymerase, and 10 pmol of each standard M13 primers: sense 5' GTAAAACGACGGCCAGTG 3' and antisense: 5' GGAAACAGCTATGACCATG 3'. The colony was taken directly from the plate and resuspended into the 20 μl PCR reaction by pipetting. 10 μl of the thermocycled PCR reaction was separated on .9% agarose stained with 10 μg/ml ethidium bromide.

### Restriction analysis colony screen

The REN digestion excision assay follows pre-established methods, using the plasmid pBKOα [32] (Fig. 9). pBKOα and a *piggyBac *expression helper plasmid are cotransfected into S2 cells and incubated. Plasmid DNA is recovered by modified Hirt extraction as detailed in the blue/white colony screen section of methods and transformed into *E. coli *cells. A portion of this recovered plasmid is digested with *Bgl*II to remove any helper and non-excised donor plasmids and plated on LB-Amp/X-gal/IPTG plates. Because excision removes the lacZ gene, white colonies are scored as excision events. A portion of the undigested Hirt extraction is also transformed and plated on LB-Amp/X-gal/IPTG plates and the number of blue colonies is scored as the total number of potential donor plasmids.

10^6 ^S2 cells were allowed to adhere to the bottom of a 9.6 cm^2 ^cell culture well and transfected with 0.5 μg of phsp-pBac helper plasmid and 0.5 μg of pBKOα donor plasmid with Transfectin liposomal transfection reagent (Bio-Rad) according to manufacturer protocol in minimal S2 media (Gibco). Two wells were transfected per helper-donor pair. The transfection media remained on the cells overnight and was replaced with Schneider's complete media. At 24 hours post transfection the cells were heat shocked for 1 hour at 37°C. At 24 hours post heat shock the cells were harvested by Hirt extraction as detailed above and resuspended in 10 μl water. At this time the two individual samples were pooled and 2 μl of the DNA sample was digested with high activity *Bgl*II restriction enzyme (NEB) for 4 hours at 37°C, ethanol precipitated as above and resuspended in 22 μl water. The entire sample was transformed by electroporation into 3 μl *E. coli *DH10B (Invitrogen) in a 1 mm gap cuvette and recovered in 75 μl S.O.C. media (Invitrogen). 50 μl of sample was immediately plated onto two LB-Ampicillin plates prepared with IPTG and X-gal and incubated at 37°C for 16 hours. White colonies were picked and cultured overnight in 3 ml LB-Ampicillin broth. The DNA was extracted by crude boiling mini-prep and digested with *Hind*III restriction enzyme (NEB) and run on a 0.8% agarose gel to screen for positive excision events.

For the background control 1 μl of the pooled Hirt extracted DNA was resuspended in 21 μl of water and transformed into 3 μl of DH10B *E. coli *and recovered in 75 μl of S.O.C. media. 5 μl of this preparation was immediately plated on two LB-Ampicillin agar plates containing IPTG and X-gal and incubated at 37°C for 16 hours and the blue colonies were counted.

### *piggyBac *antibody

5 ml of rabbit anti-serum was obtained from Proteintech Group, Inc. after injection with truncated *piggyBac *protein. The antiserum was subjected to 50% ammonium sulfate precipitation for 60 minutes at 4°C and centrifuged at 10,000 g for 60 minutes. After resuspending the ammonium sulfate pellet in 20 mM K_2_HPO_4 _pH 6.8, 50% ammonium sulfate precipitation was performed again. After centrifugation, the resulting pellet was resuspended again and underwent buffer exchange in 20 mM K_2_HPO_4 _using a Centriprep YM-50 (Fisher Scientific, St. Louis, MO) according to manufacturer's protocol. This was followed by concentration of the IgG protein fraction using a Centricon YM-50 (Fisher Scientific). The concentrated IgG fraction was further purified using a 15.5 mL DEAE Affi-Gel Blue Gel Column (Bio-Rad Laboratories, Hercules, CA) according to manufacturer's protocol. The elution buffer was 20 mM K_2_HPO_4_, 0.02% sodium azide pH 6.8. The eluted IgG anti-*piggyBac *antibody was concentrated using a Centricon YM-50 to a final concentration of 16.57 mg/mL as determined by optical density spectrophotometry at 280 nm on a Nanodrop ND-1000 spectrophotometer (Nanodrop Technologies Inc., Wilmington, DE). Anti-*piggyBac *antibody function was verified through western blotting of known positive and negative protein samples (data not shown).

### Western Blot

S2 Cells were co-transfected as described above along with two additional samples containing 1 μg of either a mutant helper, phsp-pBac, as a positive control or a non-*piggyBac *expression plasmid as a negative control. 48 hours post-induction, the cells were collected and pelleted by centrifugation at 3,000 rpm for 2 minutes. This was followed by two 1 ml washes with 1× PBS. The resulting cell pellets were lysed by resuspending in 100 μl 2× Laemmli Sample Buffer containing 10 mM benzamidine, 10 mM sodium fluoride, 100 mM sodium vanadate, 1 mM phenylmethanesulphonylfluoride (PMSF), 25 μg/ml leupeptin, 25 μg/ml aprotinin, and 25 μg/mL pepstatin. The whole cell lysates were then sonicated on ice for 15 seconds. The protein concentration was measured by optical density spectrophotometry at 280 nm on a Nanodrop ND-1000 spectrophotometer.

The whole cell lysates were prepared for electrophoresis on a 1.0 mm thick 7.5% acrylamide SDS-PAGE gel by diluting 125 μg of total protein to a total volume of 25 μl using 2× Laemmli sample buffer. This was followed by heating for 5 minutes at 95°C with 1.25 μl 2-mercaptoethanol to completely denature the protein. The samples were loaded onto two separate gels with 5 samples per gel and electrophoresed at 100V using 1× Tris-glycine plus 0.1% SDS as the running buffer. The protein standards used were Invitrogen's Benchmark Prestained protein ladder. After electrophoresis was complete, the separated proteins were transferred to a Hybond-ECL nitrocellulose membrane (Amersham Biosciences, Piscataway, NJ) using the Bio-Rad Mini Trans-Blot Cell and Module for 1 hour at 350 mAmps. 1× Tris-glycine with 20% methanol was the transfer buffer.

Following transfer, the membranes were stained with Ponceau stain and subsequently destained with 5% acetic acid and 1× PBS washes (images not shown). The blots were blocked using 1× PBS plus 5% non fat dry milk for 2 hours while shaking at room temperature. The blots were subsequently probed for *piggyBac *protein expression using a 1/1000 dilution of anti-*piggyBac*, purification described above, or for actin using anti-actin I-19 (Santa Cruz Biotechnology, Santa Cruz, CA) in a 1/500 dilution with 1× PBS plus 0.1% Tween-20 (PBS-T) and 5% non-fat milk for one hour while shaking at room temperature. After the primary incubation, the blots were washed four times for five minutes each with 1× PBS-T. The secondary antibody, ECL rabbit IgG, HRP-linked whole antibody (Amersham Biosciences) or donkey anti-goat IgG-HRP (Santa Cruz Biotechnology), respectively, was added in a 1/5000 dilution with 1XPBS-T and 5% non-fat dry milk for one hour at room temperature while shaking. This was followed by washing with 1× PBS-T four times for five minutes each. The blots were developed with Pierce's SuperSignal West Dura Extended Duration Substrate kit (Pierce, Rockford, IL) according to manufacture protocol except 0.5 ml of each substrate was used per blot.

### Statistical analysis

Data are expressed as a mean of three replicates +/- standard. ANOVA test was performed using GraphPad Prism 3.0 software. All values were adjusted to a positive control setting of 100%. Means were considered statistically significant when p-values less than 0.05 were obtained with the Dunnett's post-test. Statistical significance is indicated on all data figures as asterisks above bars.

## Authors' contributions

JHK created the donor plasmids used in the blue/white colony screen, performed all aspects of the blue/white screen, performed the sequence alignment, BoxShade processing and alignment analysis, and prepared the manuscript. CAS performed the western blot and prepared parts of the manuscript dealing with the western blot. TSF performed all aspects of the REN colony screen. MJF conceived of the study and provided guidance.
